# Bioelectronic measurement and feedback control of molecules in living cells

**DOI:** 10.1038/s41598-017-12655-2

**Published:** 2017-10-02

**Authors:** Areen Banerjee, Isaac Weaver, Todd Thorsen, Rahul Sarpeshkar

**Affiliations:** 1 Departments of Engineering, Microbiology & Immunology, Physics, and Physiology & Neurobiology, Dartmouth College, Hanover, New Hampshire 03755 USA; 2 Thayer School of Engineering, Dartmouth College, Hanover, New Hampshire 03755 USA; 3 MIT Lincoln Laboratory, Massachusetts, 02421 USA

## Abstract

We describe an electrochemical measurement technique that enables bioelectronic measurements of reporter proteins in living cells as an alternative to traditional optical fluorescence. Using electronically programmable microfluidics, the measurement is in turn used to control the concentration of an inducer input that regulates production of the protein from a genetic promoter. The resulting bioelectronic and microfluidic negative-feedback loop then serves to regulate the concentration of the protein in the cell. We show measurements wherein a user-programmable set-point precisely alters the protein concentration in the cell with feedback-loop parameters affecting the dynamics of the closed-loop response in a predictable fashion. Our work does not require expensive optical fluorescence measurement techniques that are prone to toxicity in chronic settings, sophisticated time-lapse microscopy, or bulky/expensive chemo-stat instrumentation for dynamic measurement and control of biomolecules in cells. Therefore, it may be useful in creating a: cheap, portable, chronic, dynamic, and precise all-electronic alternative for measurement and control of molecules in living cells.

## Introduction

Negative-feedback loops are important for regulation and homeostasis in biology^1–10^ and in engineering^11^ in both natural and synthetic systems. They serve to architect precise and robust control of output variables in accord with a reference set-point determined by the user. A high feedback loop gain can achieve precise control but often makes the feedback loop more prone to oscillation^12^. Strategies to prevent oscillation in feedback loops in engineering often use Proportional, Integral, Derivative (P.I.D.) or equivalent circuit compensation techniques^10^.

Until recently, control systems were used in Chemostats to control external growth conditions for cells like temperature or oxygen. However, recent advances in microfluidics have allowed us to exploit control-engineering principles to regulate molecular reactions within living cells. Prior work by Toettcher *et al*. has demonstrated an optical feedback control system in mammalian cells^13^. The ability of microfluidic-based devices to tightly control both the introduction of inducers in the system and the growth medium renders them an attractive tool for use as a tool for gene regulation^14^. Quantitative regulation of gene expression in a yeast-based system was recently demonstrated using a microfluidic platform^15–18^. Similarly, Fracassi *et al*. demonstrated a microfluidic-based feedback control strategy in mammalian systems^19^ using fluorescence based reporters. We sought to build an all-electronic feedback control system for gene expression in bacterial systems using an electrochemically sensitive molecule to serve as an output detector.

**Figure 1:**
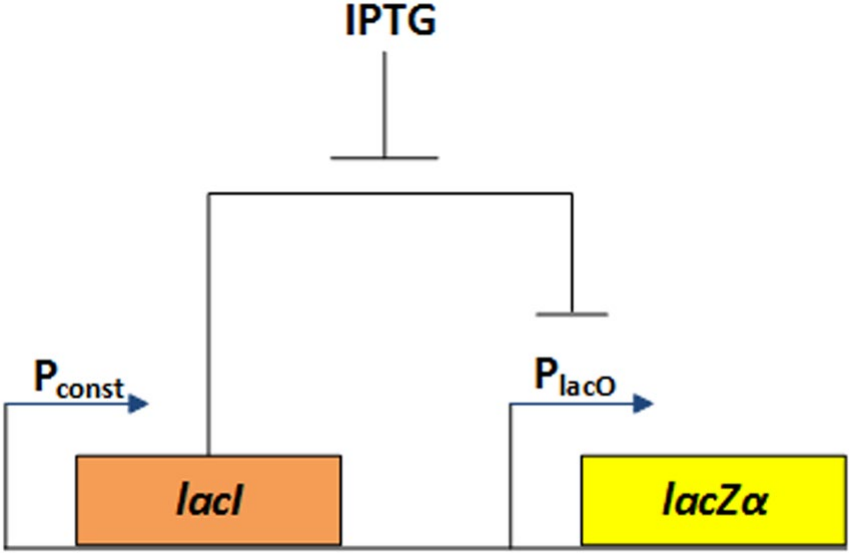
Synthetic IPTG inducible Repression circuit producing *lacZα*. A schematic of the genetic circuit with *lacZα* under the control of the *lacI*, IPTG & P_*lacO*_ system. The product of *lacI* inhibits the expression of *lacZα* by shutting down the activity of P_*lacO*_. IPTG inhibits LacI activity by binding to it and allows for expression of LacZα.

To create an alternative for optical measurements that would be precise but that would be cheaper and more portable, we describe a genetic circuit in *Escherichia coli* with an inducible promoter controlled by an exogenous input (IPTG) and an associated transcription factor (LacI) that together serve to regulate the production of a *β*-galactosidase enzyme (Fig. 1). The resulting *β-*galactosidase enzyme breaks down the Chlorophenol Red *β-*galactopyraonoside (CPRG) substrate molecule into an electrochemically sensitive dye, Chlorophenol Red (CPR) and galactose. The CPR is oxidized at an electrode to produce electrical current that is digitized and then used to control an electronically programmable lab-on-a-chip microfluidic system^20–23^. The electrical current produced has a linear relationship to the concentration of CPR (Figure S1). The microfluidic system alters the amount of inducer input thus creating a regulatory feedback loop. An electronically programmable set-point in a microprocessor associated with the microfluidic system effectively determines the regulated value of LacZ*α* in the bacterium, and the feedback dynamics can be altered by changing software parameters in the electronic control strategy.

## Results

**Figure 2:**
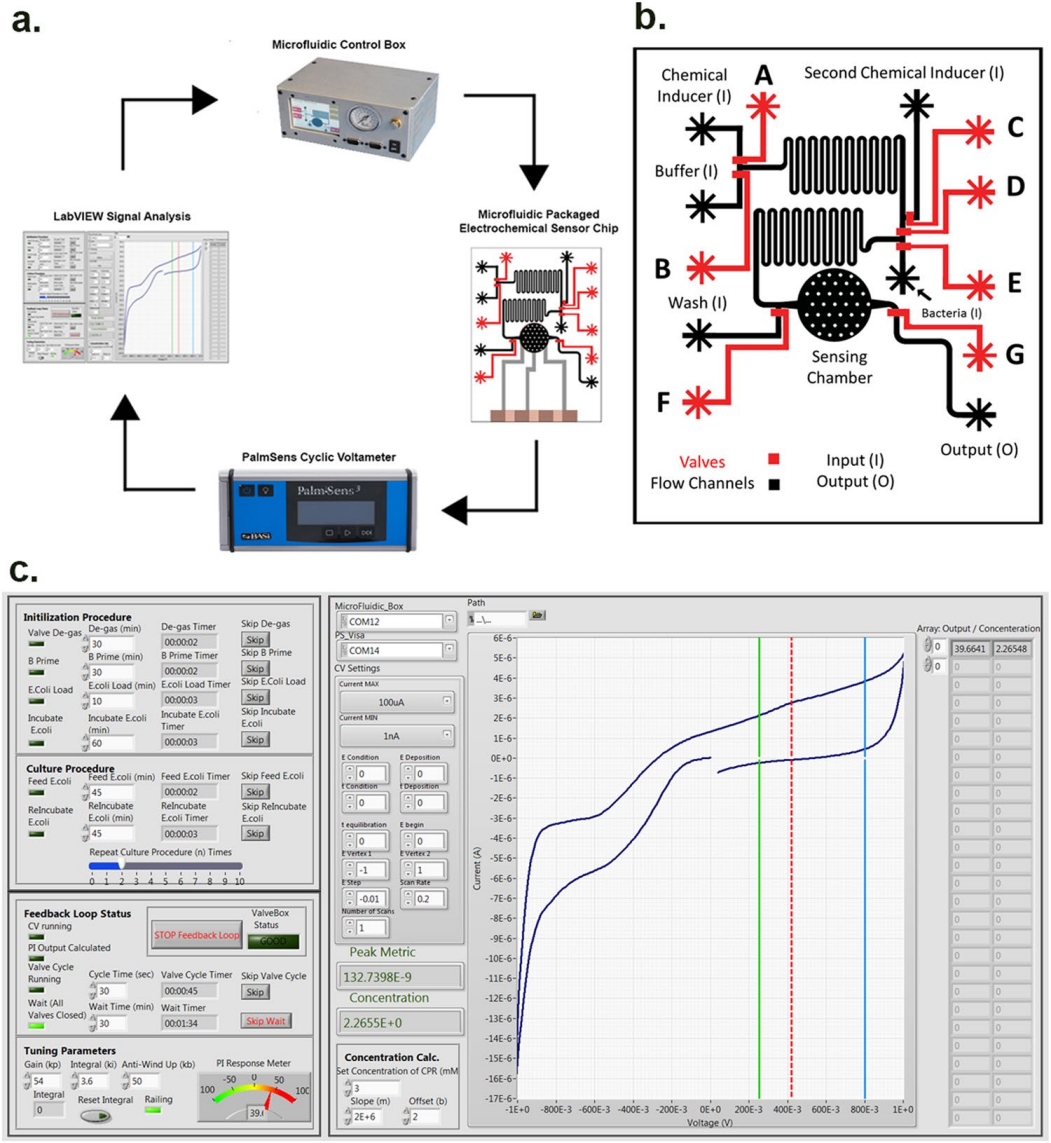
Microfluidic control system and architecture of chip. (**a**) Schematic of the microfluidic control of biological circuits. After the two inputs mix in serpentine channels, they enter the cell culture chamber underneath which are three electrodes for electrical measurements. The resulting voltage sweep data is received by the LabVIEW program that calculates the relative concentration of CPR in the chamber and instructs the microfluidic box to adjust the input to correct the system chemistry appropriately. (**b**) Architecture of the microfluidic chip. It was specially designed with two serpentine channels to promote proper mixing of inputs. (**c**) A front panel view of the LabVIEW program.

Achieving precision in communicating with a biological system requires delivery of very small amounts of inducer stimulus be given to cells. To automate biological protocols and precisely control the mixing of chemical inducers, we built a custom microfluidic electrochemical sensing “Lab-on-a-chip” (LOC) (Fig. 2a,b) along with a microfluidic control box that synchronizes with it^20–23^. We used the LOC in conjunction with a feedback loop to regulate the inducible synthetic circuit in *E. coli* NEB10*β*. The output of this synthetic circuit is measured by the level of CPR which produces an electrical signal upon oxidation at the electrode. To measure the CPR concentration and regulate the chemical input of the inducer by the microfluidic chip, we wrote a closed-loop program in LabVIEW (Block Diagram in Figure S3). We obtained the equation for determining the CPR concentration by generating a standard curve of CPR concentration vs. Area under the curve of the peak measured during Cyclic voltammetry (peak obtained between 0.4 V–0.8 V) as shown in the supplemental figures (Figure S1). For the automation of biological protocols on our custom microfluidic chips, we designed and built a portable microfluidic controller, featuring an open-source Arduino microprocessor that would implement our feedback control loop (Fig. 2a).

To calibrate the control of our electronic feedback loop, we first created a LabVIEW program for *in-vitro* CPR preparation (for calibration) which was subsequently modified for intended use in the *in-vivo* microbial system. The sampling rate for the relatively fast *in-vitro* CPR system was set to 30 seconds whereas, for the relatively slow *in-vivo* microbial feedback system it was set to 1,800 seconds. A P.I.D. control strategy was implemented for the control input *u(t*) as a function of the measured error w.r.t. the electronic set-point using Equation (1) below:

1 $$u(t)={K}_{p}e(t)+{K}_{i}{\sum }^{}e\,{\rm{\Delta }}t+{K}_{b}\frac{{\rm{\Delta }}e}{{\rm{\Delta }}t}$$

Gain (*K*_*p*_), Integral (*K*_*i*_), and Derivative (*K*_*b*_) constants were optimized experimentally with initial approximate values determined with the Ziegler-Nichols method^24^. Cycling of the microfluidic valves functions digitally with Pulse Width Modulation (PWM). To obtain the highest resolution in the ratio adjustments, the program and experiments were designed such that the ratio of Chemical Inducer (**A**) to Buffer (**B**) would be 50:50 at the onset of experimentation. Based on the subsequent measurement of CPR in the system, the PID response meter (Fig. 2c) takes corrective measures to account for deviation of measured CPR concentration from the set point. Such measures are achieved by changing the relative ratio of inducer (**A**) to Buffer (**B**). The final values used to demonstrate the feedback loop *in-vivo* were in the ratio of *54:3.6:50* for *K*_*p*_ *:K*_*i*_:*K*_*b*_ respectively (Fig. 2c).

The LabVIEW program is designed to completely automate the experiment once all the systems are set up and the inputs to the microfluidic chip are connected. The microfluidic chips use Quake valves^23^ to control the flow of fluids through the chips. The first step in the process is to outgas these valves to ensure proper actuation. Once we have achieved properly functional valves, the next step is to allow for the buffer and CPR to flow through the channels to remove any residual air. Using the new feedback loop, the system was calibrated initially with the electrochemically sensitive molecule (CPR) and a buffer solution. The program triggers the Palmsens potentiostat to run a Cyclic voltammetry sweep across the electrodes positioned under the culture chamber of the microfluidic chip. The electrodes are made from 100 nm of platinum, deposited via evaporation atop a Pyrex glass slide and subsequently patterned. Cyclic voltammetry can be prone to fouling; cleaning the electrodes during fabrication is critical to achieving consistent measurements. As the potentiostat sweeps the voltage between -1 and + 1 V, starting at 0 V, at a scan rate of 0.2 V/s, starting at 0 V, the current is measured, CPR measurements yield redox peaks which correspond to its relative concentration. We found a reliable peak to occur on the upper sweep between 400 mV and 800 mV; the program looks in this range for the peak. The bounds in which the program looks for the peak are set graphically by the green and blue bars (Fig. 2c). The program looks for the inflection point in the curve to either side of the peak. It then draws a line between these two inflection points and takes the area under the peak to the drawn line. The correlation between the redox peak and CPR concentration was externally characterized with known CPR concentrations on identical electrodes. By characterizing the linear relationship between CPR concentration and the integral of the peak current, we obtained a more accurate algorithm and implemented that into the new feedback loop. After extensive tuning, we could achieve very precise adjustments of the system using PID control.

**Figure 3:**
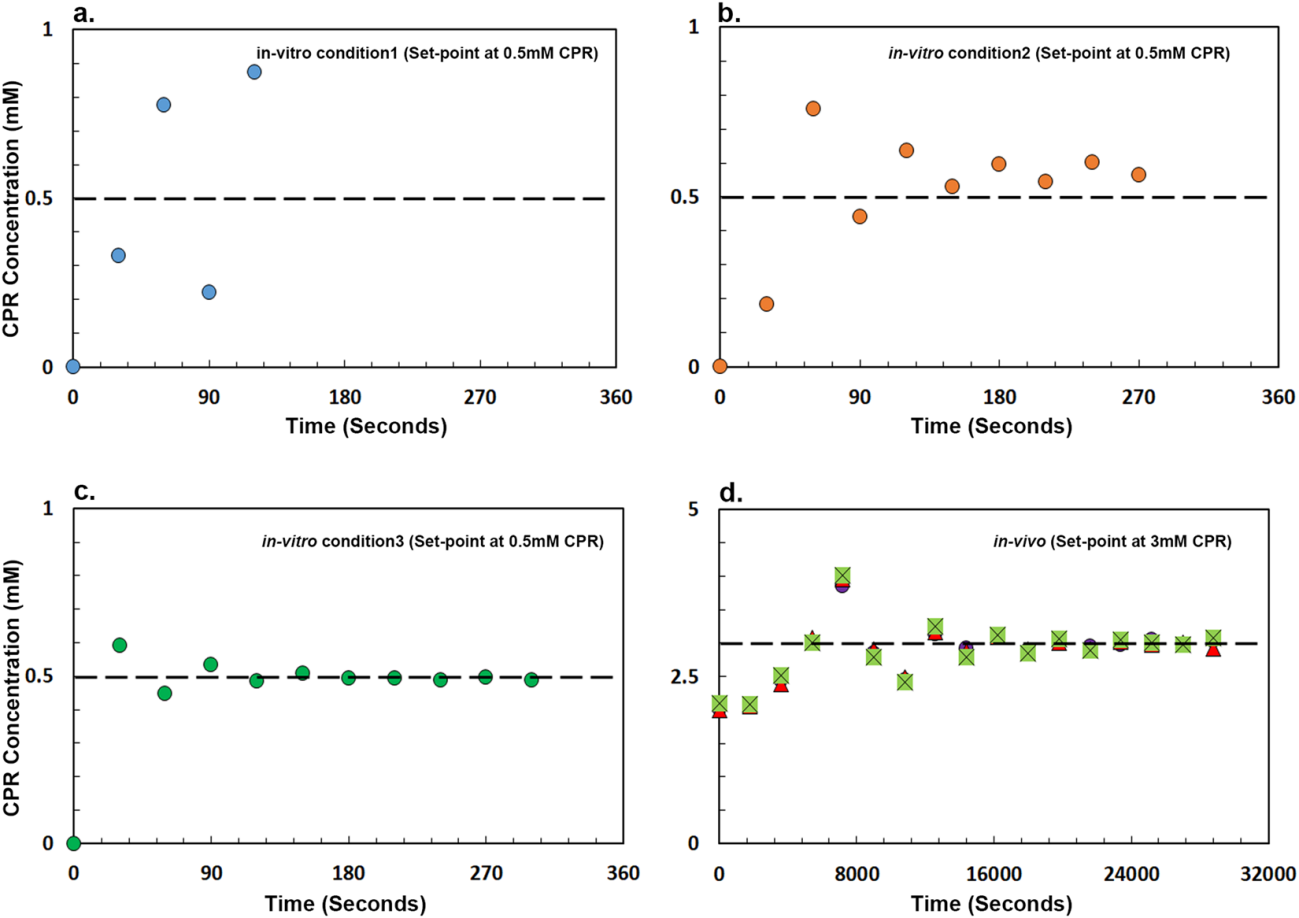
Microfluidic based feedback control of CPR dye. Tuning the feedback control with Dye and PBS buffer. The graph with blue circle (**a**) is extremely unstable and has high oscillations. After changing the parameters, the graph with brown circle (**b**) has significantly less oscillation but it has higher error and stabilized above the threshold set by us. The graph with green circle (**c**) has accurate parameters which allow us to observe the final stabilization very close to the set-point. (**d**) Describes the Microfluidic Feedback control of a genetic circuit in *E. coli*. The graphs are 3 independent experiments performed with separate starter cultures of *E. coli* and in different chips.

We observed that *in-vitro* calibration is dependent on the parameters of the control. Initial parameters (Table S1) were approximately set at the beginning of each experiment and subsequently modified during calibration. The results shown in Fig. 3a (blue graph) demonstrated high oscillation in the amount of CPR detected. After modifying the parameters (K_p_ of 50 and K_i_ of 50), it was observed that even though the output had significantly less oscillation (Fig. 3b, brown graph), there was a variation between the desired set point and the experimental set point. Finally, Fig. 3c (green graph) showed that, with appropriate parameters (K_p_ of 50 and K_i_ of 40), we could obtain both very little oscillation and low error between the desired set-point and experimental set-point.

We then sought to test this system *in-vivo*. The synthetic circuit to demonstrate our proof-of-concept system is a simple repression circuit where a LacI protein inhibits the expression of LacZα (Fig. 1). The presence of IPTG inhibits LacI activity and allows for production of LacZ*α* which can breakdown CPRG into CPR. We tested the ability of our control system to regulate the output of this genetic circuit by regulating the levels of IPTG inducer presented to the cell. Figure 3d illustrates the *in-vivo* microfluidic feedback control of l*acZα* expression in a microbial system. All three independent experiments demonstrated that the set-point defined by the microfluidic box (in this instance 3 mM CPR) controls the amount of LacZ*α* produced by *E. coli* precisely. We also confirmed that the β-galactosidase enzyme carried out the CPR degradation. We compared the measured peak area due to CPR production over time by an *E. coli* strain containing a plasmid that makes *β-*galactosidase induced with 1 mM IPTG to a strain lacking the plasmid. In the absence of *β-*galactosidase, the measured peak area is 1000x smaller (Figure S2).

**Figure 4:**
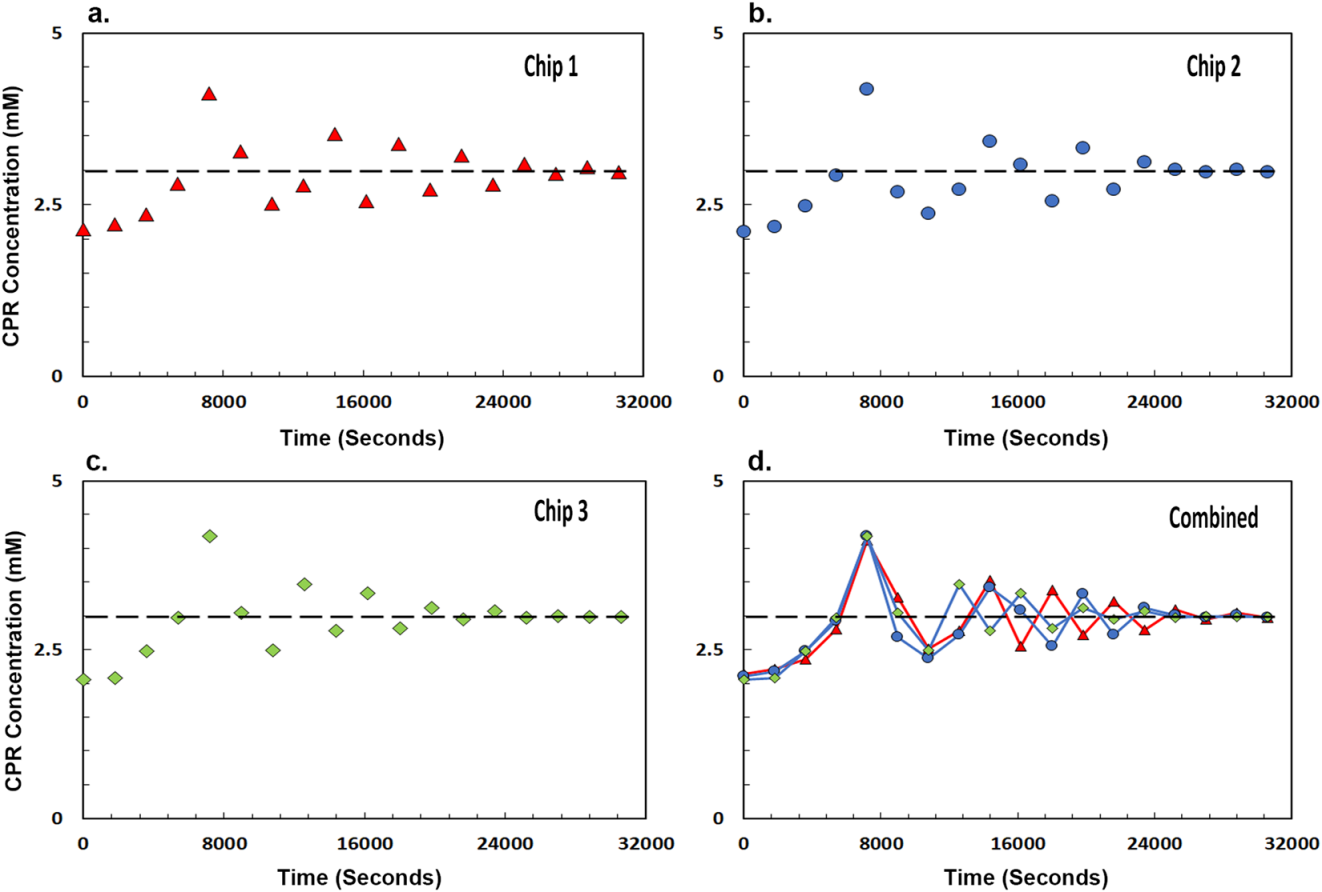
Robustness of the PDMS Microfluidic Chip design. The graphs with red triangle (**a**), blue circle (**b**) and green diamond (**c**) describe the microfluidic feedback control of the genetic circuit described in Fig. 1 performed in three independent microfluidic chips. The chips were designed using the same protocol as described in the methods section but at a different time from the chips used in Fig. 3d. (**d**) Each experiment equilibrates to the exact same set-point precisely as programmed by the user in three different chips. In Fig. 4d, lines have been drawn to avoid clutter amongst data points, thus enhancing clarity. They are not fits to the data.

We tested the robustness of the chip design process and confirmed that the feedback control could be obtained with different batches of the PDMS chips. Figure 4 showed that we can achieve similar results with different batches of microfluidic chips. Figure 4a–c illustrate graphs from three independent experiments that were performed on three different chips. These chips were designed using the same previously published procedure as the chips used in Fig. 3d ^15–18^. We observed that each experiment equilibrates to the same set-point precisely as electronically programmed by the user (Fig. 4d).

## Discussion

Feedback loops play an important role in engineering systems, be it in self-adaptive software engineering or in designing bio-reporter systems. We have described a proof-of-concept ‘LOC’-based microfluidic chip & control system that can be used to precisely control molecules in *E. coli* via a synthetic circuit. We have utilized a LacZ*α*-based electrochemical detection system that reduces the level of toxicity in comparison with typical fluorescence-based systems (*β-*galactosidase is a naturally occurring enzyme as compared to GFP which is foreign to a bacterium and is toxic when produced in large amounts)^25^. The P.I.D-based LabVIEW program was custom designed to act as an interface between a transimpedance amplifier and a microfluidic control box. The P.I.D. control strategy reduces oscillations in the system with an appropriate choice of parameter values. As a consequence of feedback-control robustness, we observe that CPR levels in the growth chamber equilibrate to a precise set-point determined by the user independent of culture or the chips that were used. Finally, we were able to architect precise negative-feedback control in a bacterial system using a breakdown of CPRG to CPR via the *β*-galactosidase enzyme.

For its potential use in industrial applications, the LacZ*α* reporter used in this proof-of-concept work can be replaced by other essential genes. Since we can achieve robust control of gene expression in synthetic circuits using electronic control, this work may have broad utility in bioengineering. Thus, may be useful in creating a cheap, portable, and precise all-electronic system for measurement and control of essential molecules in bacteria.

## Materials and Methods

### Bacterial strains and growth conditions

*Escherichia coli* Strain NEB10β (*araD139* Δ*(ara-leu) 7697 fhuA lacX74 galK (*ϕ*80 (M15) mcrA galU recA1 endA1 nupG rpsL* (Str^R^) Δ*(mrr-hsdRMS-mcrBC)*) (New England Biolabs, Table S2) was used for generation of all relevant strains for this study. *E. coli* cells were cultivated with LB medium supplemented with appropriate antibiotics when necessary at 37 °C with shaking. For microfluidic feedback testing, *E. coli* strains were grown in LB medium with antibiotics.

### DNA manipulation and plasmid construction

All enzymes for DNA manipulation were from New England Biolabs unless stated otherwise. Phusion DNA polymerase (New England Biolabs) used for all DNA amplifications, except for colony PCR where we used Qiagen *Taq* polymerase. Plasmids, PCR products, and DNA fragments from agarose gel were purified with Qiagen miniprep, PCR purification, and Gel extraction kits, respectively.

The low copy plasmid (pSC101) (Table S2) used as a backbone for inserting our circuit was obtained from the standard lab stock strains. The primers (Table S3) and parts for the synthetic circuit for expression of *lacZα* (Fig. 1) were designed using G-Blocks (IDT DNA). The plasmid backbone and parts were put together by PCR using Gibson Assembly kit (New England Biolabs). The resulting plasmid pAB007-1 (Table S2) was sequenced and confirmed to be accurate using commercially available sequencing services (Genewiz).

### Microfluidic feedback control of CPR dye

To test the microfluidic feedback control using Chlorophenol Red (CPR) dye, 3 mM CPR stock in PBS was made and filter sterilized. Microfluidic tubing (Tygon Microbore 0.020 ‘‘internal diameter × 0.060” outer diameter) was filled with 3 mM CPR and Phosphate Buffered Saline (PBS) diluent respectively and inserted into the appropriate channels. Furthermore, tubes containing water were introduced into the valves controlling the channels. These were connected to the appropriate channels in the control box. Before running the microfluidic box, the valves (tubes containing water) were outgassed for 20 minutes to remove any air bubbles. After the appropriate amount of time had elapsed, the valves were shut and the tubes containing CPR and PBS were connected. The LabVIEW program controlling the feedback loop started and data collected.

### Microfluidic control of CPRG breakdown in *E. coli*

Set up for the microfluidic chip and control box for testing *E. coli* cells was the same as described in the previous section. We grew the bacterial cultures containing the IPTG-inducible synthetic circuit overnight in LB with kanamycin as the selective antibiotic. We aspirated these cultures into the Tygon tubes (specifications mentioned in previous sections and inserted into the line controlled by ‘Valve A’. In another tube, we aspirated the diluent (LB) and CPRG (Chlorophenol Red β-galactopyraonoside) solution and put it into ‘Valve B’. A third tube was inserted into the input of ‘Valve C’ containing 1 mM IPTG. The whole rig was attached to the microfluidic control box and potentiostat device. The LabVIEW program with the PID control then allowed a predefined cell feed and growth run to seed the cells into the chip. After the cells grew for 2 hours, CPR stabilization data was collected.

### Data Availability

The data generated during and analyzed during the current study are available from the corresponding author on reasonable request.

## Electronic supplementary material

Supplementary Material
