# Supplementary material for: Bioelectronic measurement and feedback control of molecules in living cells
Source: Sci Rep. 2017 Oct 2;7:12511. doi: 10.1038/s41598-017-12655-2 (PMC5624954; doi:10.1038/s41598-017-12655-2)
Supplement: Supplementary file 1 — Supplementary Material [file 41598_2017_12655_MOESM1_ESM.pdf]

Supplementary Materials for

# Bioelectronic measurement and feedback control of molecules in living cells

Areen Banerjee, Isaac Weaver, Todd Thorsen, Rahul Sarpeshkar*

correspondence to: rahul.sarpeshkar@dartmouth.edu

**Figure S1:**

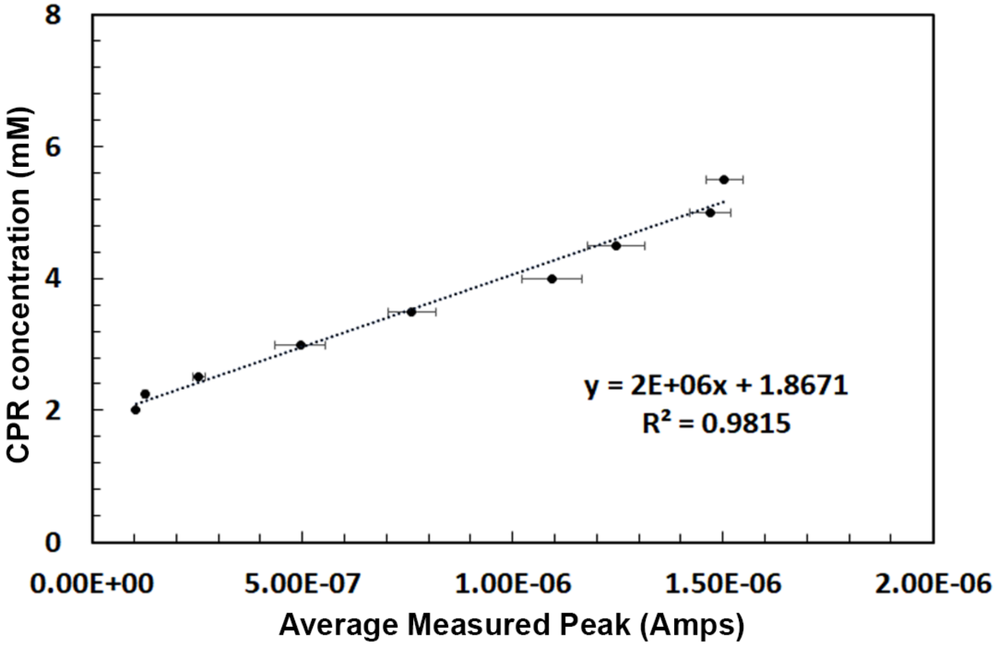


**Figure S1:** Standard curve showing the linear relationship between CPR concentration (mM) and the Area Under the peak (Amps). In the equation CPR concentration(y) is dependent on the current produced during Cyclic Voltammetry sweep by the PalmSense potentiostat.

**Figure S2:**

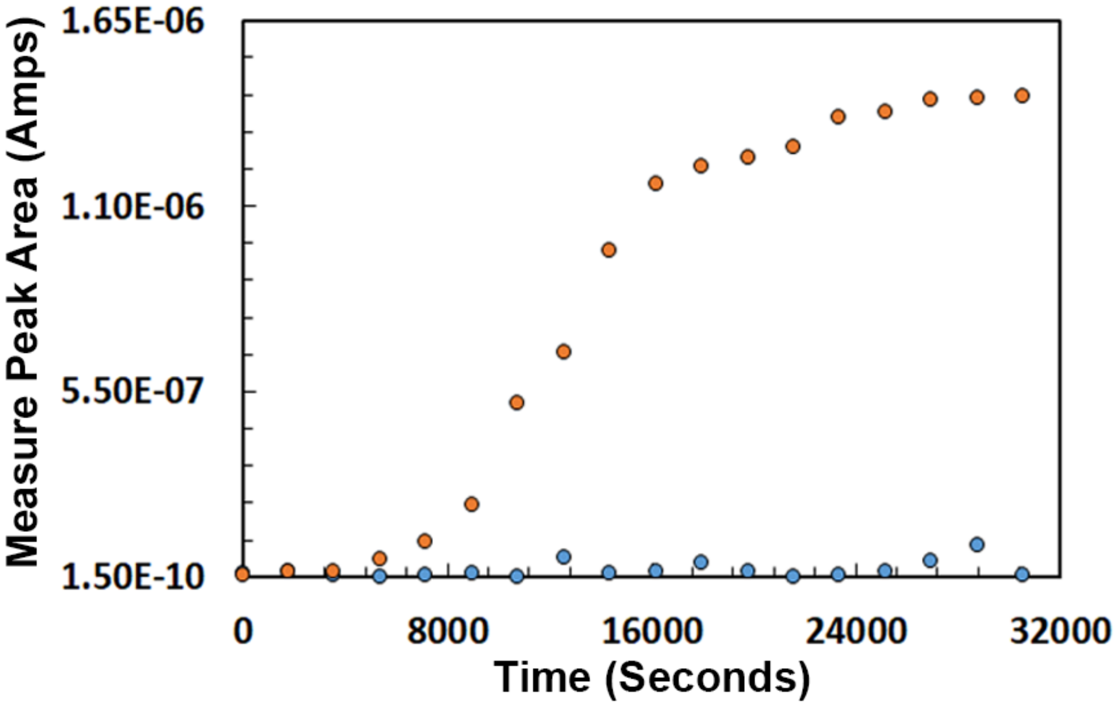


**Figure S2:** Measured peak area due to CPR production over time by *E. coli* strain containing a plasmid that makes β-galactosidase induced with 1mM IPTG (Orange circles). In the absence of any β-galactosidase, the measured peak area is $10^3$ times lower (Blue circles).

**Figure S3:**

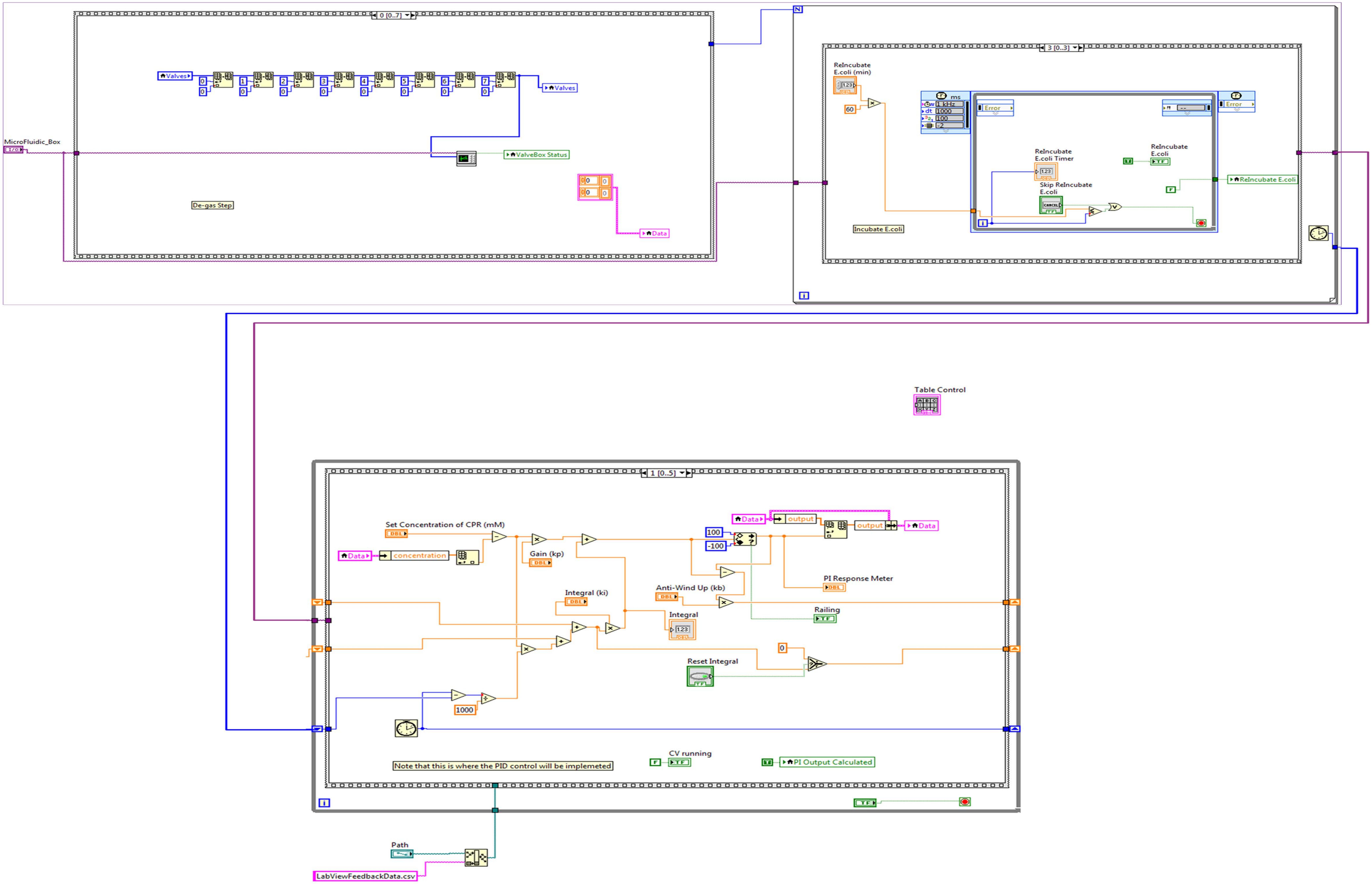


**Figure S3:** Block Diagram of the closed-loop program written using LabVIEW. The Front panel of this program with all its GUI is shown in **Figure 2c**.

**Figure S4:**

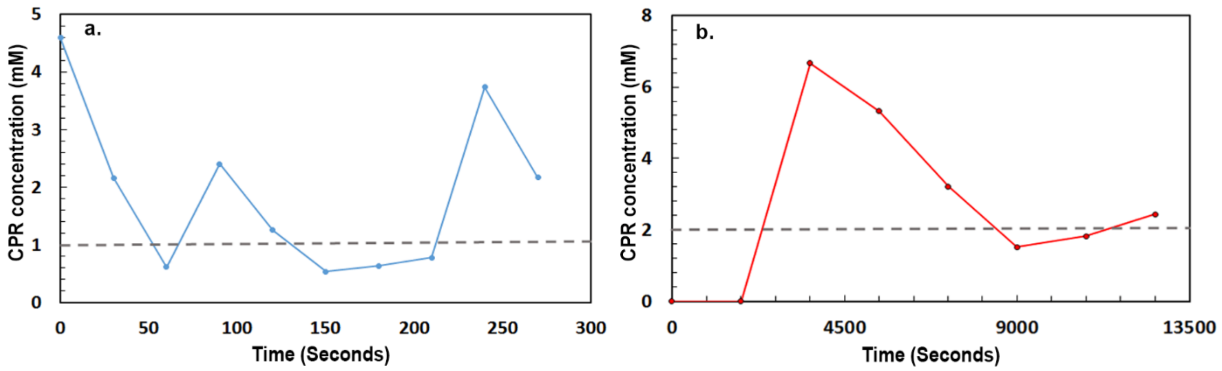


**Figure S4:** Preliminary Microfluidic based feedback control of CPR dye. **(a.)** Tuning the feedback control with Dye and PBS buffer. The blue graph is extremely unstable and has high oscillations because of the lack of PID control (The control program uses Linear control). Each time the PalmSet calculates the concentration of CPR in the system, it either adds CPR dye or adds PBS buffer. This leads to oscillation behavior but it fails to set at the threshold. **(b.)** Similar results are obtained when *E. coli* containing the simple *lacZα* expressing circuit is used to breakdown CPRG into CPR. These results were used to design a PID based system which provided us with good oscillation and final settling of the system at the set threshold (**graphs a., b. & c. from Figure 3**).

**Table S1.** Parameters set for *in-vitro* CPR feedback loop calibration.

| | **Proportional to $K_p$** | **Proportional to $K_i$** | **Result** |
|---|---|---|---|
| **Condition 1** | -3 | 10 | Large oscillation observed |
| **Condition 2** | 50 | 50 | Oscillation reduced but error in experimental vs. Manual Set-point |
| **Condition 3** | 50 | 40 | Minimal oscillation and error |

**Table S2.** Plasmids & Strains used in this study.

| **Name** | **Description** | **Source** |
|---|---|---|
| **pSC101** | Low copy plasmid containing *E. coli* replicable origin | Lab Stock |
| **pAB007** | Low copy plasmid derived from pSC101 containing synthetic circuit with IPTG inducible *lacZα* gene. | This Study |
| ***E. coli*** **NEB10β** | *araD139 Δ(ara-leu) 7697 fhuA lacX74 galK (ϕ80 M15) mcrA galU recA1 endA1 nupG rpsL* ($Str^R$) *Δ(mrr-hsdRMS-mcrBC)* | New England Biolab |
| **EC16** | *E. coli* NEB10β containing pAB007 low copy plasmid | This Study |

**Table S3.** Primers used in this study.

| Name | Sequence |
|---|---|
| **pAB007bkbnRev** | GACGTCGGAATTGCCAGC |
| **pAB007part1fwd** | GAGGGCGCCCCAGCTGGCAATTCCGACGTC TTGACGGCTAGCTCAGTC |
| **pAB007part1Rev** | AATTTGATTGCGAGTGAGATATTTATGCCA GCCAG |
| **pAB007part2Fwd** | TGGCATAAATATCTCACTCGCAATCAAATTC AG |
| **pAB007part2Rev** | TATCCGCTCACAATTAAGGATGAAGGTTAG TCTAGG |
| **pAB007part3Fwd** | CTAACCTTCATCCTTAATTGTGAGCGGATAA CAATTGAC |
| **pAB007part3Rev** | GTTTGCGGGCAGCAAAACCCGTACCCTAGG TCTAGGGCGGCGGATTTG |
| **pAB007bkbnRev** | CCTAGGGTACGGGTTTTGC |
| **PconstJ23111_Seq** | TTGACGGCTAGCTCAGTC |
| **LacI1_Seq** | GTGAAACCAGTAACGTTATAC |
| **LacI2_Seq** | CTCGCGCAACGCGTCAGTGG |
| **LacI3_Seq** | CATGTCCGGTTTTCAACAAAC |
| **PlacO_Seq** | AATTGTGAGCGGATAAC |
| **LacZ_Seq** | ATGACCATGATTACGGATTC |
